# Innovation in the percutaneous management of benign hepaticojejunostomy stricture with multiple lithiasis using SpyGlass Discover and biodegradable stent: a case report

**DOI:** 10.1093/jscr/rjaf878

**Published:** 2025-11-06

**Authors:** Edison Santiago Borja Villacrés, Jefferson Andrés Carvajal Torres, Santiago Andrés Muñoz-Palomeque

**Affiliations:** Department of Surgery, Hospital de los Valles, Quito, 170902, Ecuador; Department of Surgery, Clínica Internacional, Quito, 170508, Ecuador; Pontificia Universidad Católica del Ecuador, Quito, 170143, Ecuador; Universidad Tecnológica Equinoccial, Quito, 170147, Ecuador; Department of Radiology, Hospital Vozandes, Quito, 170521, Ecuador; Department of Surgery, Hospital Metropolitano, Quito, 170508, Ecuador; Faculty of Medical, Health and Life Sciences, Universidad Internacional del Ecuador, Quito, 170411, Ecuador

**Keywords:** hepaticojejunostomy, bile duct stricture, percutaneous cholangioscopy, lithotripsy, biodegradable stent, spyglass

## Abstract

We report the case of a 51-year-old female with a history of iatrogenic bile duct injury treated with hepaticojejunostomy in 2013 and reconstruction in 2016. She developed recurrent anastomotic stricture and intrahepatic lithiasis, with multiple failed surgical and endoscopic attempts, for that reason a two-stage percutaneous approach was performed: initial transhepatic cholangiography, crossing the stricture area and placing an internal-external biliary catheter; followed by cholangioscopy through a mature tract using the Spyglass Discover system, electrohydraulic lithotripsy of intrahepatic stones, endoluminal balloon plasty, and placement of biodegradable stent fifteen days later. The patient achieved complete clearance of intrahepatic stones, stricture resolution, and normalization of liver biochemistry without early recurrence. This case highlights the feasibility and efficacy of a minimally invasive percutaneous strategy combining cholangioscopy, lithotripsy, and biodegradable stenting for complex benign hepaticojejunostomy strictures, even in nondilated bile ducts, avoiding the morbidity of open surgery.

## Introduction

In recent years, the number of patients with hepaticojejunostomy anastomotic (HJA) strictures has increased [1]. Benign hepaticojejunostomy strictures (HJS) most commonly occur following iatrogenic bile duct injury, liver transplantation, or pancreatic surgery [2]. Traditional treatment relies on open surgical reconstruction [3, 4], or in centers with endoscopic gastroenterology, performing an enterotomy on the efferent loop to allow retrograde introduction of the endoscope and dilation of the anastomosis is another option.

Although surgical revision remains the reference standard, it is associated with notable morbidity and a risk of mortality that can reach 10% in complex cases. Recurrence occurs in ~15%–25% of patients, often requiring repeated interventions [3, 4]. Each new operation also increases the technical complexity, as progressive fibrosis and ductal shortening limit the available hepatic ducts for reconstruction [4].

An alternative consists of laparotomy to locate the efferent limb, enterotomy, and endoscopic dilation of the anastomosis from within in an attempt to avoid redoing the anastomosis. However, it still carries the risks inherent to open surgery (anastomotic leak, adhesions, hernia, and sepsis) and may require repeated laparotomies if recurrence occurs [5, 6]. These approaches often require multiple interventions and extended hospital care, adding to the overall treatment burden for both patients and healthcare systems [7].

Given the high rates of morbidity/mortality and the economic burden of open surgery, minimally invasive techniques based on percutaneous and endoluminal access have been developed over recent decades to treat both strictures and intrahepatic stones with less tissue trauma [2, 8]. The following case exemplifies this shift toward percutaneous approaches.

## Case report

A 51-year-old female presented with recurrent cholangitis and jaundice. She had sustained a Strasberg type D bile duct injury during a laparoscopic cholecystectomy in 2012, treated with HJA in 2013 and open reconstruction in 2016. A new stricture recurrence occurred in 2021, treated with transjejunal endoscopic dilation assisted by open surgery. In 2023, she developed a stricture recurrence with episodes of cholangitis and multiple intrahepatic lithiasis. A percutaneous approach was attempted; however, according to the report, it failed due to the lack of biliary tract dilation, so the patient was referred to our team.

**Figure 1:**
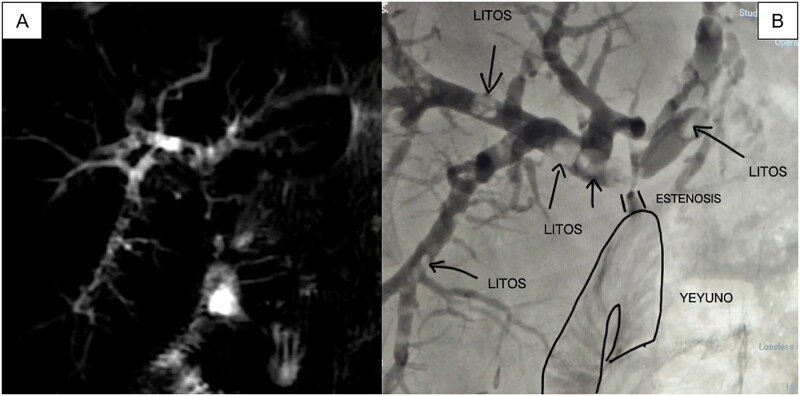
(A) MRI showing multiple filling defects in the right and left main branches, as well as stenosis at the level of the HJA; (B) Initial cholangiography obtained by puncturing the peripheral branch of segment VI with a 21-gauge needle confirms the same findings as the MRI.

Magnetic resonance cholangiopancreatography (MRI) revealed HJA stricture without significant biliary dilation (left hepatic duct 6.2 mm, right 5.5 mm) and four intrahepatic stones (4–5 mm) (Fig. 1A). Laboratory values showed total bilirubin (TB): 3.36, direct bilirubin (DB): 3.11; aspartate aminotransferase (AST): 74, alanine aminotransferase (ALT): 63.

We carried out a two–stage procedure. During the stage 1 procedure, a percutaneous transhepatic cholangiography (THPC) was performed via segment VI. Despite nondilated ducts, the duct was cannulated, and multiple filling defects (lithiasis) were observed, as well as threadlike passage of contrast toward the efferent loop (Fig. 1B). Recanalization techniques successfully traversed the stricture, and an internal-external biliary catheter (8 Fr) was placed. The procedure lasted 90 minutes, and the patient was discharged the next day without complications.

**Figure 2:**
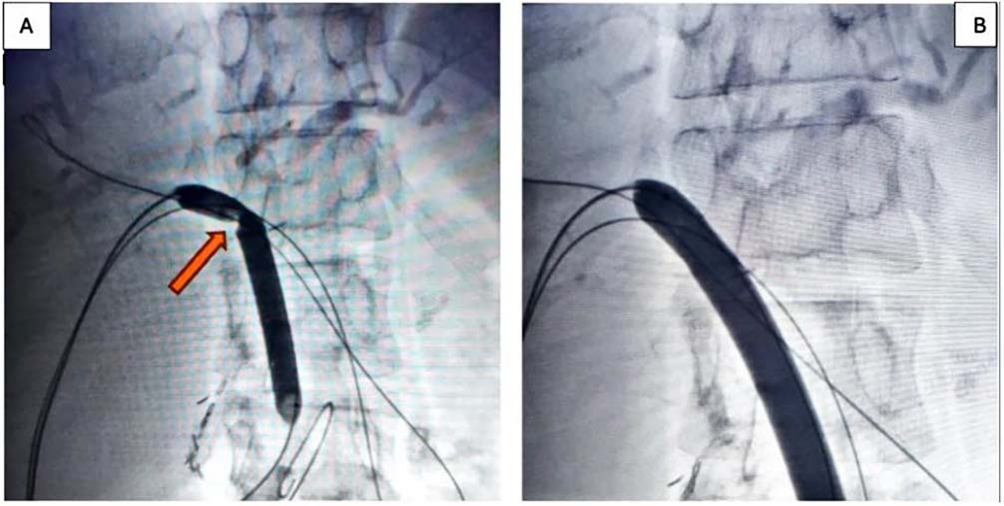
(A) 10 × 60 mm percutaneous balloon fully inflated at the anastomosis level (arrow shows the notch formed at the stenosis level); (B) Projection after performing endoluminal plasty for 3 minutes, maintaining the same inflation pressure.

**Figure 3:**
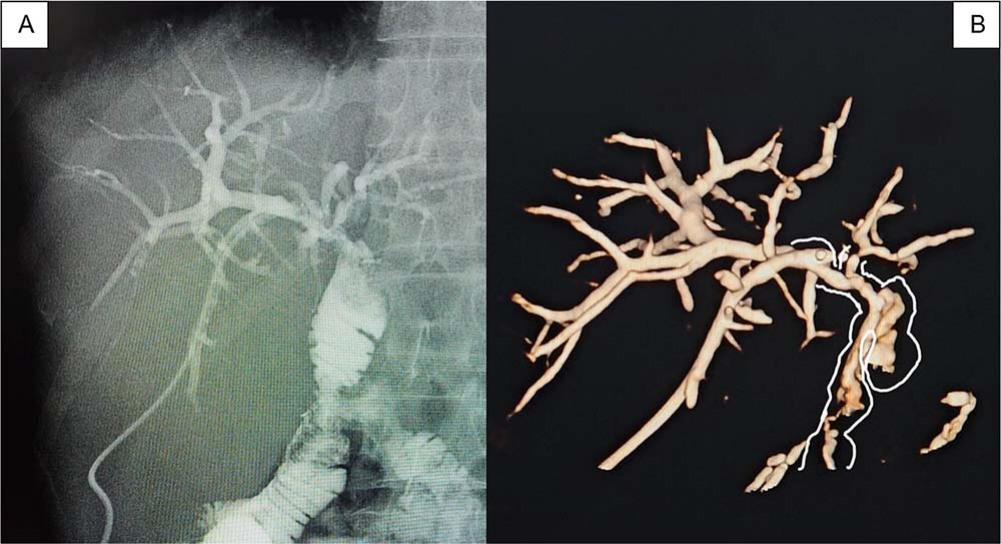
(A) Pre-removal cholangiography of a Nelathon catheter placed in the peripheral branch of segment VI to ensure the path in case of complications, showing fluid passage toward the efferent loop; (B) 3D tomographic reconstruction allows a 360-degree view of the passage of contrast throughout the biliary tree.

In the stage 2 procedure (15 days later), Spyglass Discover cholangioscopy was performed using the matured tract. Cholangiography was performed through the catheter, identifying the persistence of intrahepatic stones. The catheter was removed, and using the Seldinger technique, the fistulous tract was progressively dilated to 13 Fr to place an 11/13 Fr Navigator introducer. Through this, the single-operator Spyglass Discover - Boston Scientific choledochoscope was advanced, navigating toward the right and left main hepatic branches. Electrohydraulic lithotripsy (EHL) was applied to the stones, completely fragmenting them. Subsequently, endoluminal plasty of the anastomosis was performed with a 10 × 60 mm balloon (Fig. 2A and 2B), and a 10 × 57 mm biodegradable stent was implanted. Irrigation is then performed with 300 milliliters of saline solution to sweep the stone fragments toward the jejunal loop. The final cholangiography demonstrated adequate contrast flow throughout the right and left biliary trees and toward the loop (Fig. 3A). An 8 Fr feeding tube was left in segment VI to secure the path in case of complications with the stent and was left connected to a free-fall collection bag. Total procedure time was 180 minutes.

The patient recovered uneventfully. A control cholangiography with 3D tomographic biliary reconstruction was performed, demonstrating adequate functioning of the stent (Fig. 3B); therefore, it was decided to remove the catheter from the fistulous tract to allow spontaneous closure by secondary intention. The patient had a 24-hour hospital stay. At control, 30 days post-procedure, normalization of the cytolytic profile was observed: TB: 0.98, DB: 0.89, patient asymptomatic.

## Discussion

HJS occur in about 7.4% of patients with a preoperative bile duct diameter less than 8.8 mm [9]. This case demonstrates the evolving role of minimally invasive techniques in complex benign HJS management. Historically, the management of a stenosis of HJA has evolved from open techniques to minimally invasive techniques, with a notable evolution as we describe and summarize in (Table 1) [4–6].

**Table 1:** Comparative and evolutionary table of the main strategies for treating benign HJA stricture and intrahepatic lithiasis, with their advantages, disadvantages, success rates, and recurrence

**Strategy**	**Advantages**	**Disadvantages/complications**	**Success (patency)**	**Restenosis**	**References**
Open surgery (HJ revision)	High technical success; allows resection of scar tissue	High morbidity ~20% (bile leak 4.5%, surgical-site infection [SSI] 10%), mortality ~2%; prolonged length of stay; high costs	87%	13%	[3, 4]
Prolonged external drainage	Simple procedure; avoids surgery in the acute phase	Causes dehydration, coagulopathy, dyspepsia; electrolyte imbalance; limits activity and quality of life	Does not restore anatomy	Not resolved	[2, 8]
Percutaneous balloon dilatation	Minimally invasive; available in many centers; ~10% complication rate	Requires three sessions; possible anastomotic dehiscence; prolonged catheter dependence	82%–88% at 3 years	18.8%–35%	[2, 11]
Sustained dilatation with multiple catheters	Maintains continuous dilatation	Risk of suture rupture and retained fragments; risk of multiple re-interventions	89%–96.7%	9.4%–11.4%	[2, 5, 6]
Uncovered metallic stent	Maintains initial luminal caliber	High risk of hyperplasia and tissue ingrowth; makes future surgery difficult because removal is impossible	65%	35%	[2, 8]
Covered metallic stent (partial or full)	Prevents hyperplastic tissue ingrowth	Migration 4%–31%; biloma due to occlusion of secondary branches; may require exchange	80%–91%	8%–11%	[1, 2]
Biodegradable stent	No removal required; allows orderly epithelial growth	Higher cost than catheters; variable degradation; requires implantation expertise; risks include partial anastomotic dehiscence 2%, hemobilia 4%, migration 2%	85%–95%	18% (year 1); 27% (5 years)	[10, 11]
SpyGlass + EHL + biodegradable stent (present case approach)	Percutaneous access, even in a nondilated biliary tree; enables targeted lithotripsy and precise dilatation; minimal morbidity; short hospital stay (1–2 days); avoids prolonged catheters	Requires availability of a cholangioscope and percutaneous expertise; possible cholangitis (~4.5%)	Success 95.4%	18% (year 1); 27% (5 years)	[10, 12, 13], Present case

Denial of percutaneous approach due to lack of dilation was common since it was previously considered that the biliary tract should have a minimum diameter of 6–8 mm to allow safe percutaneous access [8]. This belief led many patients to undergo extensive open surgeries, with high morbidity and prolonged stays (average 36 days) [3, 4]. The described case demonstrates that percutaneous transhepatic cannulation is feasible even in 5.5–6.2 mm ducts with the aid of microguides and microcatheters [2, 8].

Biodegradable stents represent a promising alternative, maintaining dilation while degrading naturally, thus avoiding reinterventions for removal. Studies report long-term patency rates of 79%–87% with reduced recurrence compared to balloon dilation alone [10, 11].

Since 2022, Spyglass DS direct visualization system-assisted percutaneous transhepatic cholangioscopy has been described as a safe, feasible, and effective procedure for the diagnosis and treatment of biliary diseases in patients with surgically altered anatomy, particularly in those with the Roux-en-Y reconstruction requiring a percutaneous approach [12, 13].

The addition of Spyglass Discover cholangioscopy allowed targeted EHL for intrahepatic lithiasis clearance, with a reported complication rate of <5% [12]. This combination addresses both stricture and lithiasis in a single minimally invasive session, minimizing hospital stay and avoiding repeat surgery [2, 8].

## Conclusion

Percutaneous management using Spyglass cholangioscopy, electrohydraulic lithotripsy, and biodegradable stenting provides a safe, effective, and minimally invasive alternative to open surgical revision for complex benign HJS with lithiasis, even in nondilated ducts <6 mm. This approach reduces morbidity, shortens hospitalization, and offers durable outcomes.

Compared to open or laparoscopic surgery, which retains its role in select cases, percutaneous procedures should be considered first-line when adequate human and technological resources exist. The notion that the non-dilated bile duct cannot be treated percutaneously is disproved; evidence shows that with high-quality ultrasound and fluoroscopic support, ideally an angiograph, high expertise, and fine guidewires with appropriate dilators, it is feasible and safe. The implementation of biodegradable stents and visualization systems such as Spyglass, along with the availability of a percutaneous surgeon or interventional radiologist in tertiary hospitals, allows for the resolution of complex complications of hepatobiliary surgery with a minimally invasive, cost-effective approach and low morbidity.
